# Improving Individual Brain Age Prediction Using an Ensemble Deep Learning Framework

**DOI:** 10.3389/fpsyt.2021.626677

**Published:** 2021-03-23

**Authors:** Chen-Yuan Kuo, Tsung-Ming Tai, Pei-Lin Lee, Chiu-Wang Tseng, Chieh-Yu Chen, Liang-Kung Chen, Cheng-Kuang Lee, Kun-Hsien Chou, Simon See, Ching-Po Lin

**Affiliations:** ^1^Aging and Health Research Center, National Yang Ming Chiao Tung University, Taipei, Taiwan; ^2^NVIDIA AI Technology Center, NVIDIA, Taipei, Taiwan; ^3^Institute of Neuroscience, National Yang Ming Chiao Tung University, Taipei, Taiwan; ^4^Center for Geriatrics and Gerontology, Taipei Veterans General Hospital, Taipei, Taiwan; ^5^Brain Research Center, National Yang Ming Chiao Tung University, Taipei, Taiwan

**Keywords:** structural MRI, neuroimaging, brain age, machine learning, ensemble deep learning, regularization

## Abstract

Brain age is an imaging-based biomarker with excellent feasibility for characterizing individual brain health and may serve as a single quantitative index for clinical and domain-specific usage. Brain age has been successfully estimated using extensive neuroimaging data from healthy participants with various feature extraction and conventional machine learning (ML) approaches. Recently, several end-to-end deep learning (DL) analytical frameworks have been proposed as alternative approaches to predict individual brain age with higher accuracy. However, the optimal approach to select and assemble appropriate input feature sets for DL analytical frameworks remains to be determined. In the Predictive Analytics Competition 2019, we proposed a hierarchical analytical framework which first used ML algorithms to investigate the potential contribution of different input features for predicting individual brain age. The obtained information then served as *a priori* knowledge for determining the input feature sets of the final ensemble DL prediction model. Systematic evaluation revealed that ML approaches with multiple concurrent input features, including tissue volume and density, achieved higher prediction accuracy when compared with approaches with a single input feature set [Ridge regression: mean absolute error (MAE) = 4.51 years, *R*^2^ = 0.88; support vector regression, MAE = 4.42 years, *R*^2^ = 0.88]. Based on this evaluation, a final ensemble DL brain age prediction model integrating multiple feature sets was constructed with reasonable computation capacity and achieved higher prediction accuracy when compared with ML approaches in the training dataset (MAE = 3.77 years; *R*^2^ = 0.90). Furthermore, the proposed ensemble DL brain age prediction model also demonstrated sufficient generalizability in the testing dataset (MAE = 3.33 years). In summary, this study provides initial evidence of how-to efficiency for integrating ML and advanced DL approaches into a unified analytical framework for predicting individual brain age with higher accuracy. With the increase in large open multiple-modality neuroimaging datasets, ensemble DL strategies with appropriate input feature sets serve as a candidate approach for predicting individual brain age in the future.

## Introduction

The trajectory of healthy brain aging is characterized by a complex dynamic process with progressive and regressive changes in brain structure and function (1–3). Previous group level neuroimaging studies have identified potential relationships between aging processes and regional characteristics of the brain (2, 4–6) and suggested that these aging-related alterations in the human brain may be associated with the incidence of several neurodegenerative diseases (7, 8). Therefore, the method of obtaining aging-related bio-signatures with higher reliability based on different brain characteristics is of particular importance.

In the last decade, the novel concept of “biological brain age” has emerged and served as a candidate quantitative index for assessing individual brain health throughout the entire lifespan (6, 9). Several studies have estimated brain age using extensive neuroimaging data from healthy participants with different machine learning (ML) approaches (10, 11). Furthermore, several public health-orientated studies have demonstrated the potential interrelationships between individual brain age and mortality risk, grip strength, and physical activity (9, 12). Several clinically oriented studies have supported the clinical relevance of brain age in several neurodevelopmental and neurodegenerative disorders, including Alzheimer's disease (10, 13), schizophrenia (14, 15), and traumatic brain injury (11). Additionally, an individual's biological brain age has been proposed as a prognostic indicator for treatments and interventions in several neurological diseases (16–18). Although the concept of biological brain age has been widely applied in neuroscience, public health, and clinical research, the optimal approach to construct predictive models of brain age with higher reliability and accuracy remains a challenge.

In addition to conventional ML approaches, several end-to-end deep learning (DL) analytical frameworks have recently been proposed as alternative approaches with significant potential for predicting individual brain age and disease classification with higher prediction accuracy (19, 20). Compared with previous conventional ML-based brain age estimators, these end-to-end DL approaches omit various image preprocessing steps and feature extraction procedures which are highly dependent on software package selection and image quality. Several DL-based studies have demonstrated the superior predictive performance of this approach using single imaging modalities as the input feature set for estimating individual brain age with minimal image preprocessing procedures and feature extraction steps (19, 21, 22). However, different imaging modalities of brain MRI are associated with distinct tissue properties and provide rich information for the characterization of individual brain changes across the entire lifespan (5, 23). Ensemble learning is an effective general-purpose ML paradigm that combines prediction of individual models to achieve better performance (24, 25). Using the ensemble learning approach, different brain MRI imaging modalities can be seamlessly unified into a single predictive model while reducing overfitting and improving predictive performance. Nevertheless, the optimal approach to select and assemble appropriate input feature sets for DL analytical frameworks remains to be determined.

In the Predictive Analytics Competition (PAC) 2019 which aimed to develop the best predictive brain age model from healthy subjects based on structural magnetic resonance imaging (sMRI) data, we explored the possibility of an ensemble DL-based framework for predicting individual brain age. The two objectives of the competition were: (1) to accomplish the smallest mean absolute error (MAE) for predicted brain age and (2) to accomplish the smallest MAE while maintaining the Spearman correlation between the predicted brain age difference (calculated as predicted brain age minus chronological age) and chronological age below 0.1. To achieve these two objectives, we first investigated the potential contribution of different input feature sets to predict individual brain age with two widely used conventional ML approaches. This empirical evidence served as a baseline comparison for the subsequent ensemble DL-based predictive model. We subsequently constructed two distinct ensemble DL-based brain age models with multiple input feature sets and objective-specific regularization functions to obtain acceptable simulation results in this timely competition.

## Methods

### Structural MRI Data and General Image Preprocessing

The dataset of the 2019 PAC contained original T1-weighted structural MRI brain images from 2,640 subjects with correct age labels (https://www.photon-ai.com/pac2019). All T1-weighted brain scans were visually assessed for scan quality. Images with apparent image artifacts or gross brain abnormalities including trauma, tumors, and hemorrhagic or infarct lesions were excluded by two experienced researchers. This quality screening procedure excluded 157 participants from subsequent image preprocessing. The final training data consisted of 2,483 participants that encompassed a wide age range from 17 to 90 years. To obtain an unbiased brain age estimator and evaluate its generalizability, we randomly allocated the provided training set (*N* = 2,483, age = 36.41 ± 16.37 years, age range = 17–90 years, 1,150 males) into a training sample (*N* = 2,198, age = 36.39 ± 16.33 years, age range = 17–90 years, 1,012 males) and a hold-out validation sample (*N* = 285; age = 36.52 ± 16.74 years, age range = 18–90 years, 138 males). An additional 660 subjects without age labels formed an independent external dataset for the final benchmarking. The results of this external dataset determined the final challenge scores from two distinct perspectives mentioned above.

To investigative the interrelationships between different input feature sets and prediction performance of the brain age estimator, we used the recently proposed enhanced Diffeomorphic Anatomical Registration Through Exponentiated Lie Algebra voxel-based morphometry (DARTEL-VBM) analytical pipeline to extract multiple input feature sets including gray matter volume (GMV, modulated gray matter segments), gray matter density (GMD, unmodulated gray matter segments), white matter volume (WMV, modulated white matter segments), and white matter density (WMD, unmodulated white matter segments) information from original T1-weighted anatomical scans of each individual. This modified DARTEL-VBM approach which integrated enhanced subcortical tissue probability maps produced more accurate subcortical tissue segmentation results when compared with the original VBM approach (26). The detailed enhanced DARTEL-VBM analytical pipeline has been documented in our previous clinical study (27). The entire image processing pipeline was performed using Statistical Parametric Mapping software (SPM12, version 7487, Wellcome Institute of Neurology, University College London, UK) using MATLAB (R2016a, Mathworks, Natick, MA). Finally, the individual Montreal Neurological Institute (MNI) space GMV, GMD, WMV, and WMD as well as native space and MNI space T1-weightd images (only for ensemble DL framework) were used as candidate input feature sets for subsequent brain age prediction analysis.

### Additional Feature Extraction Strategies for Conventional Machine Learning

To reduce computation costs and avoiding overfitting, we used two additional feature extraction strategies for ML-based brain age prediction models. First, following previous parcel-wise predictive analytical studies, we used a predefined composite brain atlas to extract the average GMV and GMD of each region of interest (ROI) from the preprocessed input features sets. This composite brain atlas included 400 cortical regions based on Schaefer's functional parcellation (28) and 42 subcortical and cerebellar structures from the Harvard-Oxford subcortical atlas and spatially unbiased infratentorial template (29). This feature extraction strategy yielded 442 × 2 = 884 structural features from both GMV and GMD as input feature sets for the subsequent ML-based predictive analyses of brain age.

In addition to the parcel-wise feature extraction strategy, we applied multivariate spatial independent component analysis (sICA) and spatial regression analysis as a secondary feature extraction strategy to obtain corresponding input feature sets across study participants. The details of the sICA-based feature extraction procedure have been described in our previous work (30, 31). Briefly, the preprocessed MNI space GMV, GMD, WMV, and WMD maps of the training dataset were concatenated as 4D datasets, respectively. For unbiased comparison with the aforementioned parcel-wise approach, the Multivariate Exploratory Linear Optimized Decomposition into Independent Components (MELODIC; FSL v5.0.9; http://fsl.fmrib.ox.ac.uk/fsl/fslwiki/) tool was applied for each original input feature set to decompose concatenated 4D dataset into 400 spatially distinct components (voxel-by-component) with corresponding weighted parameters (component-by-subject) in the training sample. Subsequently, we applied spatial regression analysis of the 4D GMV, GMD, WMV, and WMD datasets against corresponding unthresholded 400 IC maps to calculate the final integrity scores (beta weights) of each IC (32). This sICA-based feature extraction strategy yielded 400 × 4 = 1,600 integrity scores from both GM and WM as input feature sets for the subsequent ML-based predictive analyses of brain age.

### Construction of Brain Age Model From Conventional Machine Learning and Deep Learning Frameworks

#### Conventional Machine Learning Framework

Two widely used conventional ML algorithms, namely ridge regression and support vector regression (SVR), were first applied to investigate the interrelationships between different input feature sets and predictive performance of the constructed brain age estimators (33). Ridge regression is an L2-norm regularization linear regression approach which penalizes the magnitude of coefficients of input features and prevents overfitting during model fitting (34). In contrast, SVR is a kernel-based regression algorithm which transforms input data from the original space into a high-dimensional space with a specific kernel function (35, 36). In this study, we used the radial basis function (RBF) kernel which is effective for modeling the nonlinear relationship between input features across training samples. Ridge regression and SVR were performed using the scikit-learn library (37). In the training sample, we applied a nested 10-fold cross-validation scheme to determine the optimal regularization parameter (λ for ridge regression) and hyperparameters (C and gamma parameter for SVR) of each ML algorithm in the inner loop and then evaluated the predictive performance of the constructed brain age estimators in the outer loop (38, 39). Specifically, in the inner 10-fold cross-validation loop, we selected the λ parameter from among five values (0.001, 0.01, 1, 10, and 100) for ridge regression and selected the C parameter and gamma parameter individually from among seven values (0.001, 0.01, 0.1, 1, 10, 100, and 1,000) for SVR to obtain the optimal parameter for each ML algorithm. For each inner 10-fold cross-validation loop, the parameters of each ML algorithm were optimized using GridSearchCV function with the “neg_mean_absolute_error” scoring parameter in the scikit-learning package (40). In the outer 10-fold cross-validation loop, we constructed the brain age estimators with the optimal parameters to estimate individual brain age. In the proposed ML-based framework, we systematically evaluated the predictive performance of the constructed brain age estimators with different combinations ranging from two feature extraction methods (parcel-wise and sICA), four input feature sets (GMV, WMV, GMD, and WMD), and two ML algorithms (ridge regression and SVR). After selecting the optimal parameters for each potential combination for the training sample, the entire training sample was used to construct the final ML-based brain age estimators which were then applied to the hold-out validation sample to evaluate the generalizability of the constructed ML-based brain age predictive models. Notably, these regression-based approaches were subject to the phenomenon of “regression toward the mean” (41). To account for this phenomenon and meet the criteria of Objective 2, we performed an additional chronological age-brain age bias correction in the conventional ML framework to adjust the predicted brain age of each individual (9). These individual residualized brain ages were used as inputs for Objective 2.

#### Ensemble Deep Learning Framework

**Figure 1:**
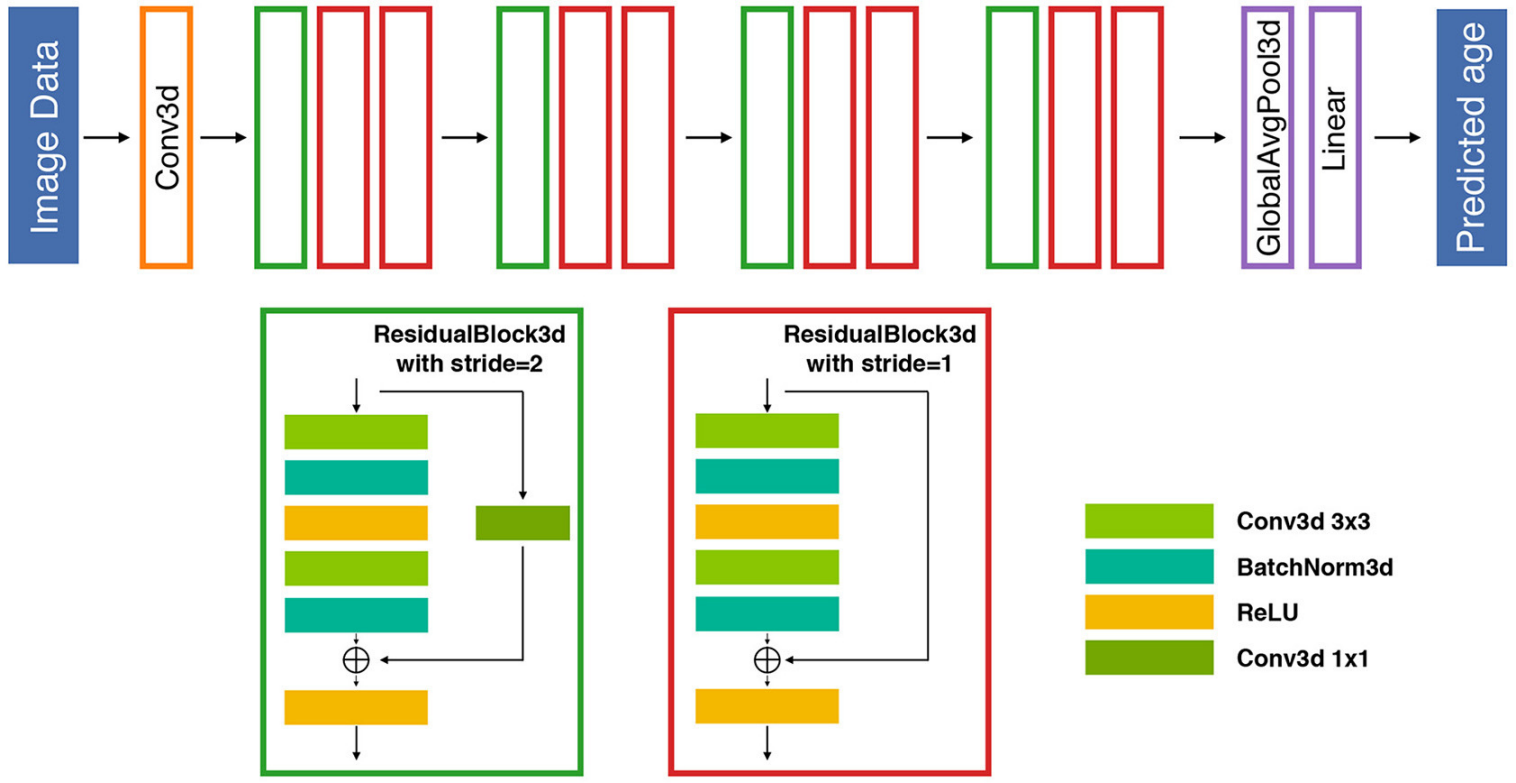
An illustration of the proposed 26-layer residual architecture for the competition. The proposed DL-based prediction model is composed of 12 residual blocks followed by a global average pooling and a fully connected linear layer to map the latent space information to individual predictive brain age. Each residual block includes two 3D convolutional layers and a residual shortcut. The stride 2 blocks reduce the output resolution of width, height, and depth to half of its inputs. An additional 1 × 1 convolution is also deployed in the shortcut to match the behavior of stride 2 blocks. BatchNorm, batch normalization; Conv, convolutional; Globalavgpool, global average pooling.

We proposed the ensemble DL framework using 26 layers of the 3D residual neural network (ResNet) composed of 3D convolution blocks by stacking a 3 × 3 × 3 convolution operation, ReLU activation function (42), batch normalization layer (43), and dropout technique (44) to construct the brain age predictive models (Figure 1). Previous studies have indicated data augmentation approach might expand the diversity of data properties and further improve the prediction performance (45–47). Therefore, to achieve superior prediction performance of the constructed ensemble DL model, we also generated novel synthetic assisted T2-weighted fluid-attenuated inversion recovery (FLAIR) images as an additional input feature set for the proposed DL models. The assisted T2-weighted FLAIR images were synthesized using an in-house U-Net generator that was trained from the BraTS dataset (https://www.med.upenn.edu/cbica/brats2019/data.html). The detailed methods of the synthesized T2-weighted FLAIR images are presented in Supplementary Methods. During this competition, the pretrained generator was applied to synthetic assisted T2-weighted FLAIR images from given raw T1-weighted images of each individual. Furthermore, we applied two regularization techniques as the loss function in the brain age model for two objectives in the competition. For the first regularization method, we used covariance matrix minimization between the error of predicted brain age *Y*′ to ground truth *Y* from chronological age:

(1) $$\begin{array}{r} L_{cov}=\;\frac{1}{N}\sum_{i=1}^{N}\left({\left((Y_{i}\prime -Y_{i})⊗Y_{i}^{T}\right)}^{2}\right) \end{array}$$

where *L*_*cov*_ is the covariance loss function; *Y*′ ∈ *R*^*N*^ is the *N* dimensional column vector containing an individual predicted brain age where *N* is the number of samples in the mini-batch; *Y* ∈ *R*^*N*^ is similar to *Y*′ but contains individual chronological age; ⊗ is the outer product; and *T* is the transpose operation on the vector.

The second regularization method for the loss function minimized the ranking relationship in each mini-batch, the minimum correlation bias of predicted brain age *Y*′, and chronological age Y:

(2) $$\begin{array}{r} L_{rank}=\;\frac{1}{N}\sum_{i=1}^{N}\left({\left((Y_{i}\prime ⊗{Y^{\prime }}_{i}^{T})-\left(Y_{i}⊗Y_{i}^{T}\right)\right)}^{2}\right) \end{array}$$

where *L*_*rank*_ is the ranking relationship loss function; *N*, *Y*′, *Y*, and *T* follow the same definitions as those in Eq. (1).

Based on the systematic evaluation of the ML approaches, we separately deployed two ensemble DL models for two objectives. In the data preprocessing, individual raw T1-weighted images and corresponding MNI space T1-weighted images were rescaled to the intensity range with [0, 1] by dividing the maximum value within the whole training data. All multi-modality brain images were further reshaped into the image dimensions of 121 × 145 × 121 with voxel size of 1.5 mm and stacked into a 4D input structure by using full-image processing. Furthermore, the DL-based brain age prediction models were constructed using a stochastic gradient descent (SGD) (48) optimizer with the following parameters: learning rate of 0.1, weight decay of 0.0005, and momentum of 0.9. The learning rate decays by a factor of 10 at 50 and 75% of the training progress. All models served for Objective 1 and 2 were trained in a total epoch of 300 and batch size of 8.

For the ensemble DL model of Objective 1, we assembled five different models including: (1) three channel inputs (raw T1-weighted images, GMV, and WMV), (2) three channel inputs (raw T1-weighted images, GMV, and WMV) with second regularization method, (3) three channel inputs (raw T1-weighted images, GMV, and WMV) with additional sex information, (4) three channel inputs (MNI space T1-weighted images, GMD, WMD), and (5) four channel inputs (MNI space T1-weighted images, GMD, WMD, and assisted T2-weighted FLAIR images) on the last layer before the fully connected regressor. We preserved the model by using the checkpoint with the smallest MAE during training. The predicted brain age was assembled by median aggregation from five distinct DL models.

For the ensemble DL model of Objective 2, the dropout rate was applied in the DL model to obtain the relative unbiased predictions. Consequently, the single 4D input structure features (raw T1-weighted images, GMV, and WMV), three different dropout rates (0.1, 0.15, and 0.2), and two different regularization methods within 300 epochs were used to construct the DL-based model. We further ranked the model from lowest to highest according to the MAE (below 3.8 years) and Spearman correlation (lower than 0.1) of 1800 checkpoints (6 configurations × 300 epochs) and selected the top eight models as the final set of predictive models for the competition. Finally, the predicted brain age was assembled by median aggregation from the eight DL models. The whole training sample was used to construct these two DL-based brain age estimators and subsequently applied to the hold-out validation sample to evaluate the generalizability of the DL-based brain age predictive models.

### Assessment of Prediction Performance

For Objective 1, the predictive performance of the constructed brain age models was evaluated using multiple quantitative indices including mean absolute error (MAE), root mean square error (RMSE), and coefficient of determination (*R*^2^) between predicted brain age and chorological brain age of the hold-out validation sample. The Kullback–Leibler divergence (KLD) was calculated as a measure to quantify the difference between the probability distributions of chronological age and predicted brain age of the hold-out validation sample.

For Objective 2, the bias prediction was evaluated using the Spearman rank correlation (Spearman's rho) between the predicted brain age difference and the chronological age of the hold-out validation sample.

## Results

### Predictive Performance of Brain Age Estimators Using Conventional Machine Learning Approaches in the Training Sample

In the conventional ML frameworks, we first demonstrated that the predictive performance of sICA-based brain age estimators was generally superior to that of parcel-based brain age estimators irrespective of tissue volume and density information as input feature sets. This result suggested that a data-driven feature extraction strategy was superior to a knowledge-driven approach for predicting individual brain age. Within each conventional ML framework, our results demonstrate that the predictive performance of the constructed brain age estimator combining multiple input feature sets outperformed those with a single input feature set. When considering the single input feature set for predicting individual brain age, the brain age estimator which used individual GMD maps as the input feature set achieved better predictive performance than the feature set of the estimator using GMV maps (Table 1). Using the sICA feature extraction strategy with four distinct feature sets (GMV, WMV, GMD, and WMD), the final constructed brain age estimator exhibited the best performance for predicting individual brain age in the training sample (ridge regression: MAE = 4.50 years, *R*^2^ = 0.88; SVR: MAE = 4.20 years, *R*^2^ = 0.94). These empirical results served as baseline conditions for comparing the results of ensemble DL approaches.

**Table 1:** Exploring the prediction accuracy of the cross-validation in the training sample using conventional machine learning frameworks.

**FE**	**Model**	**Input data**	**MAE (years)**	**RMSE**	***R*^**2**^**
Parcel-wise	Ridge	GMV	6.74	8.40	0.74
		GMD	5.84	7.40	0.79
		GMV + GMD	5.38	6.83	0.83
	SVR	GMV	5.95	7.73	0.78
		GMD	5.36	6.98	0.82
		GMV + GMD	5.04	6.56	0.84
sICA	Ridge	GMV	5.30	6.72	0.83
		GMD	4.99	6.34	0.85
		GMV + GMD	4.75	6.00	0.86
	SVR	GMV	5.10	6.52	0.84
		GMD	4.93	6.35	0.85
		GMV + GMD	4.58	5.83	0.87
sICA	Ridge	GMV + GMD + WMV + WMD	4.50	5.66	0.88
	SVR	GMV + GMD + WMV + WMD	4.20	5.38	0.89

*FE, feature extraction; GMD, gray matter density; GMV, gray matter volume; MAE, mean absolute error; RMSE, root mean squared error; R^2^, coefficient of determination; sICA, spatial independent component analysis; SVR, support vector regression; WMD, white matter density; WMV, white matter volume*.

### Predictive Performance of Brain Age Estimators Using Ensemble Deep Learning Approaches in the Training Sample

Based on the results of the conventional ML-based brain age estimators, we used multiple input feature sets to construct five distinct DL-based brain age estimators with different combinations. The final individual predicted brain age ensembled using the outputs of five distinct DL-based brain age estimators with the median aggregation approach. The predictive performance of the proposed ensemble DL Model 1 was superior to that of other sICA-based ML approaches (DL: MAE = 2.81 years, *R*^2^ = 0.94; Table 2).

**Table 2:** Exploring the prediction accuracy of the cross-validation in the training sample using deep learning frameworks.

**Model**	**Input data**	**MAE (years)**	**RMSE**	***R*^**2**^**
Ensemble DL Model 1	1. Raw T1 + GMV + WMV	2.81	4.01	0.94
	2. Raw T1 + GMV + WMV + Second regularization method			
	3. Raw T1 + GMV + WMV + Gender			
	4. MNI-T1 + GMD + WMD			
	5. MNI-T1 + GMD + WMD + assisted T2-FLAIR			

*DL, deep learning; GMD, gray matter density; GMV, gray matter volume; MAE, mean absolute error; RMSE, root mean squared error; R^2^, coefficient of determination, SVR, support vector regression; WMD, white matter density; WMV, white matter volume*.

### Generalizability of Brain Age Estimators to the Validation Sample

**Figure 2:**
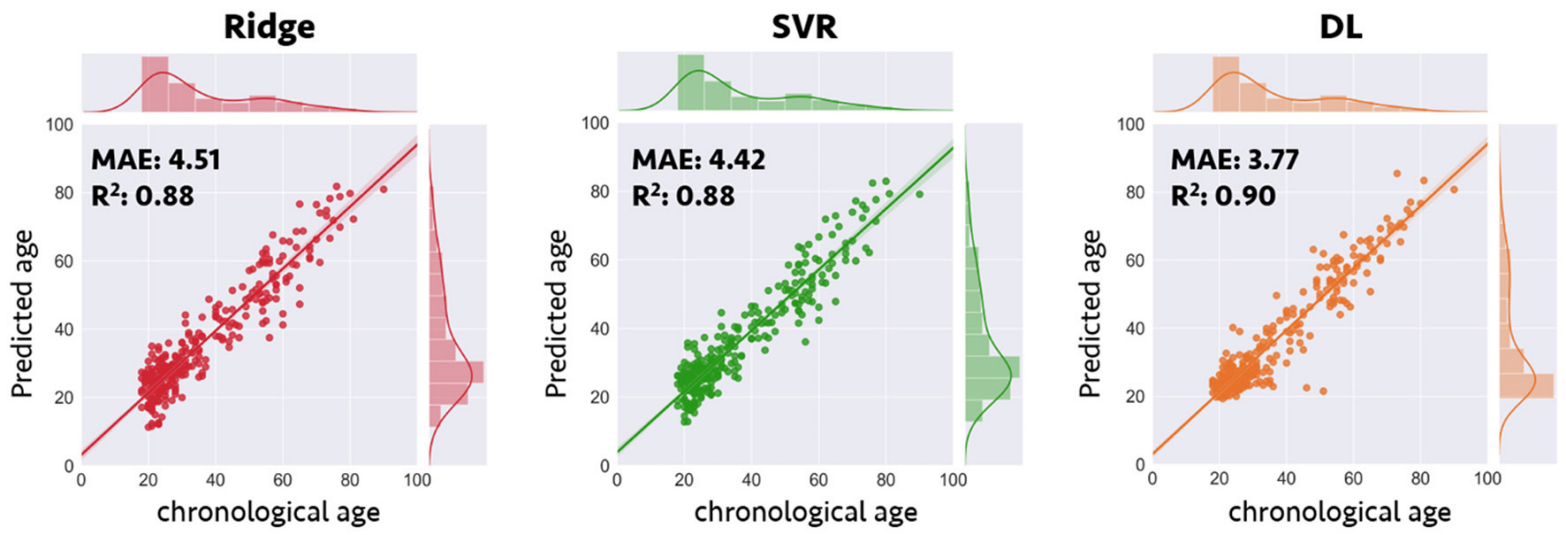
Prediction accuracy in validation sample for conventional machine learning and deep learning frameworks. Scatterplots depict the detailed data distribution of predicted brain age and chronological age for the validation sample. DL, deep learning; MAE, mean absolute error; *R*^2^, coefficient of determination; SVR, support vector regression.

**Figure 3:**
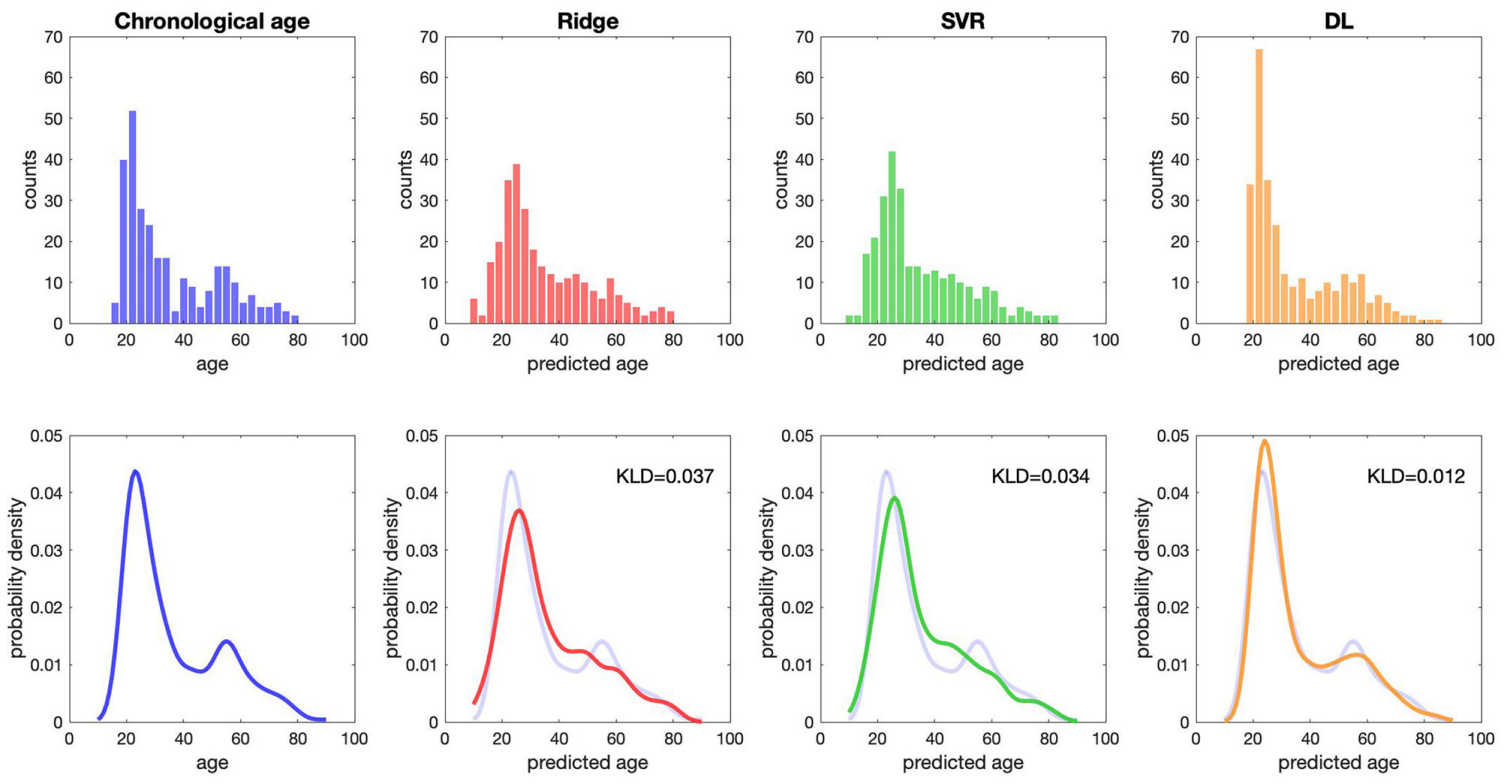
The distribution of predicted age and chronological age. The raw data distribution of chronological age and three predicted ages is presented at the top. Each bin shows the number of subjects. The overlapping distribution between chronological age and three predicted ages is presented at the bottom. DL, deep learning; KLD, Kullback–Leibler divergence; SVR, support vector regression.

Compared with the results of conventional ML approaches that used multiple input feature sets with sICA feature extraction strategy (ridge regression: MAE = 4.51 years, *R*^2^ = 0.88; SVR: MAE = 4.42 years, *R*^2^ = 0.88), the results in the validation sample suggested that each single DL-based brain age estimator provided more accurate predictions when compared with the conventional ML approaches (Model 1–1, MAE = 3.42 years; Model 1–2, MAE = 3.54 years; Model 1–3, MAE = 3.86 years; Model 1–4, MAE = 3.38 years; Model 1–5, MAE = 3.38 years), and ensemble DL Model 1 exhibited satisfactory generalizability to the validation sample (MAE = 3.77 years and *R*^2^ = 0.90) (Figure 2). In addition, using KLD as a measure for quantifying the distance between the density distribution of individual predicted brain age and chronological age, ensemble DL Model 1 produced more precise one-to-one correspondence when compared with conventional ML approaches (ensemble DL Model 1: KLD = 0.0125; ridge regression: KLD = 0.037; SVR: KLD = 0.034), especially in the validation sample with limited middle-to-late adulthood data (Figure 3).

### Brain Age Bias of Different Brain Age Estimators

The prediction accuracy of with/without bias-adjustment in the validation sample is presented in Table 3. The predicted brain age with Cole's bias correction method weakened the association between predicted brain age differences and chronological age in conventional ML and DL approaches (ridge regression: −0.0247; SVR: −0.049, ensemble DL Model 1: −0.057). However, ensemble DL Model 2 which used the covariance loss function and ranking relationship loss function as regularization methods provided accurate predictive performance for minimizing the correlation and achieving the smallest MAE (ensemble DL Model 2: −0.01).

**Table 3:** Bias correction in validation sample.

	**Without bias-adjustment**		**With bias-adjustment**	
	**MAE**	**Spearman's rank correlation**	**MAE**	**Spearman's rank correlation**
Ridge	4.51	−0.255	4.77	−0.027
SVR	4.42	−0.327	4.68	−0.049
Ensemble DL Model 1	3.77	−0.378	3.94	−0.057
	**MAE**		**Spearman's rank correlation**	
Ensemble DL Model 2	3.80		−0.01	

*DL, deep learning; MAE, mean absolute error; SVR, support vector regression*.

### Application of Brain Age Estimators to an External Testing Dataset

After systematic evaluations, we first trained two distinct ensemble DL models which targeted two different objectives of the 2019 PAC using the whole training dataset and further applied the constructed brain age estimators to an external testing dataset. For Objective 1 which aimed for the smallest MAE between individual predicted brain age and chronological age, the predictive performance of ensemble DL Model 1 was a MAE of 3.33 years with Spearman's rank correlation of −0.39. For Objective 2 which aimed for the smallest MAE while concurrently maintaining the Spearman correlation below 0.1, the predictive performance of ensemble DL Model 2 was a MAE of 3.94 years and Spearman's rank correlation of −0.013. In conclusion, we ranked the fourth place from a total of 79 teams in both objectives in the 2019 PAC.

## Discussion

In this study, we first provided empirical evidence of the possible relationship between different input feature sets and predictive performance of brain age estimation under the conventional ML-based framework. More specifically, we demonstrated that an sICA feature extraction strategy that integrated multiple features exhibited superior performance at predicting individual brain age than the parcel-wise approach. On the other hand, compared with conventional ML approaches, ensemble DL frameworks which integrated with multiple input feature sets and objective-specific regularization functions demonstrated superior predictive performance while concurrently minimizing the MAE and the correlation with chronological age in the same analytical framework.

In general, the basic analytical steps of constructing brain age estimation model included image preprocessing, feature extraction, and algorithm selection. Each step may have substantial influences on the predictive performance of the constructed brain age estimator. For T1-weighted images, multiple structural features, including tissue volume, tissue density, deformation field, cortical thickness, and surface area could be derived using different image preprocessing pipelines. Although GMV and cortical thickness of the human brain are the two most common input features for constructing brain age prediction models (10, 11, 49), previous studies also indicated that the changes in GMD play a specific role in both the developmental and aging period (5, 50). This also implies that GMD could serve as a potential candidate feature for predicting individual brain age. Our systematic evaluation also demonstrated that the prediction performance of the brain age prediction model using GMD was better than that using GMV. On the other hand, different image modalities of the brain MRI could capture the specific tissue properties of the human brain further related to the aging-associated patterns (51–53). In line with previous multimodal brain age studies, our results also support the notion that the predictive performance of the constructed brain age estimator which combined multiple input feature sets could outperform those with a single input feature set (53, 54).

Additionally, to reduce computation cost and overfitting problem in the conventional ML-based predictive framework, the feature extraction procedure was considered as an important element in predictive individual brain age. The advantage of the extracted features exerted a large impact on the performance of the prediction model (55). Compared with the use of a predefined atlas for feature extraction, the use of data-driven methods such as ICA enabled us to identify the large-scale network-wise structural covariance pattern of the structural MRI across study participants. The structural covariance is one way to measure large-scale brain morphometrical coordination profiles by estimating the similarity of tissue morphometrical features between different brain regions across participants (56). This approach is based on the notion that brain regions which interconnect with each other tend to be synchronized in maturation in a similar way, possibly due to shared neurotrophic and genetic factors (57). Using this analytical approach, previous studies also demonstrated that these identified large-scale structural covariance patterns of the human brain are highly associated with different neuropsychiatric disorders, neurodegenerative diseases, and the healthy aging process (32, 58–60). In line with previous brain age study which mainly focused on middle-to-late adulthood (31), the current study also demonstrated that the sICA-based feature extraction strategy could identify meaningful large-scale structural covariance patterns for estimating individual brain age with higher prediction accuracy.

Although the selection of the ML algorithm may affect the predictive performance of brain age estimation (51), the improvement of prediction performance was limited in this study. Potentially, the effective strategy of feature extraction method and input multimodality feature could lead to superior prediction performance, even using a relatively simpler ML algorithm. To sum up, the optimal brain age prediction, through the understanding of data characteristics and selection of machine learning strategy, could improve prediction ability. However, limitations of ML must be considered. For nonuniformly distributed data with insufficient data on middle-to-late adulthood, brain age estimation using the conventional ML-based approaches failed to achieve more accurate brain age prediction. Overcoming data bias stemming from insufficient data is an important issue for brain age estimation that should be addressed in the future. Increasing size of datasets, modifying training strategies, or using domain adaptation are possible strategies to resolve this problem.

The conventional ML algorithms may experience difficulties in engineering features to extract meaningful representations as model inputs. This process often heavily leverages prior knowledge from domain experts. However, even carefully designed feature engineering, dimensional reduction, and the loss of information may be hard to balance and are nontrivial issues to consider when seeking the optimal solution. In contrast, DL leverages the gradient descent algorithm to automatically search for a series of nonlinear transformations for feature extraction (61), which is more efficient and has the ability to obtain the optimal representation from the least preprocessed raw input data for a specific task. Additionally, in combination with ensemble learning, DL-based brain age estimation achieved superior predictive performance (62, 63). Our results also demonstrate that with a well-trained brain age model, DL could improve predictive accuracy and decrease prediction bias. Furthermore, DL was efficient at handling large-scale datasets because of the first-order gradient descent optimization algorithm. Although we demonstrated that the ensemble DL framework exhibited superior predictive performance when compared with the ML-based framework, the design of the DL framework has scope for further refinements. The model design space is extensive, and DL experts typically design based on their own experience. A recent AutoML pipeline (64) may reduce the required effort and automatically determine the optimal model. The framework of DL in brain age prediction or other neuroimaging analyses requires the concerted effort and collaborations of neuroscientists and data scientists.

## Conclusion

In summary, ensemble DL-based brain age prediction models which combined multiple input feature sets and objective-specific regularization functions provide more accurate predictive performance, decrease the bias of chronological age, and maintain correspondence even with insufficient data. Our study provides valuable insight into ML approaches and DL frameworks in brain age prediction. Our findings may facilitate the development of training strategies for brain age prediction models in the future.

## Data Availability Statement

Requests to access the datasets should be directed to Tim Hahn (hahnt@wwu.de) and Ramona Leenings (leenings@uni-muenster.de).

## Author Contributions

C-YK, T-MT, P-LL, C-WT, C-YC, L-KC, C-KL, K-HC, SS, and C-PL contributed to the conception, design, and interpretation of data. C-YK, P-LL, and K-HC performed the image preprocessing and conventional machine learning approaches. T-MT, C-WT, C-YC, and C-KL performed the deep learning approaches. C-YK and P-LL contributed to the creation of the figures. C-YK, T-MT, P-LL, C-KL, and K-HC participated in drafting the manuscript. All authors have read and approve of the final version of the manuscript.

## Conflict of Interest

The authors declare that the research was conducted in the absence of any commercial or financial relationships that could be construed as a potential conflict of interest.
