# Supplementary material for: Improving Individual Brain Age Prediction Using an Ensemble Deep Learning Framework
Source: Front Psychiatry. 2021 Mar 23;12:626677. doi: 10.3389/fpsyt.2021.626677 (PMC8021919; doi:10.3389/fpsyt.2021.626677)
Supplement: Supplementary file 1 [file Data_Sheet_1.docx]

Improving individual brain age prediction using an ensemble deep learning framework

Chen-Yuan Kuo^1†^, Tsung-Ming Tai^2†^, Pei-Lin Lee^3^, Chiu-Wang Tseng^2^, Chieh-Yu Chen^2^, Liang-Kung Chen^4,5^, Cheng-Kuang Lee^2^, Kun-Hsien Chou^3, 6, *^, Simon See^2^, and Ching-Po Lin^1,3,4,6^

^1^Department of Biomedical Imaging and Radiological Sciences, National Yang Ming

University, Taipei, Taiwan

^2^NVIDIA AI Technology Center, NVIDIA

^3^Institute of Neuroscience, National Yang Ming University, Taipei, Taiwan

^4^Aging and Health Research Center, National Yang Ming University, Taipei, Taiwan

^5^Center for Geriatrics and Gerontology, Taipei Veterans General Hospital, Taipei, Taiwan

^6^Brain Research Center, National Yang Ming University, Taipei, Taiwan

***Correspondence**:

Kun-Hsien Chou

dargonchow@gmail.com

Brain Research Center, National Yang Ming University, 155, Sec. 2, Linong St. Taipei 11221, Taiwan, +886-2-2826-7338

**^†^These authors are co-first authors**

Supplementary Methods:

This image synthetics model was first introduced by our research team in The Brain Tumor Segmentation (BraTS) challenge of 2018 which aimed to provide superior segmentation performance for tumor localization. In order to expand the variety of structural image features in predicting individual brain age for the 2019 PAC, we decided to generate synthetic assisted T2-weighted FLAIR images as an additional input feature set for the proposed DL-based brain age predictive model. The detailed information for synthesized T2-FLAIR images was listed as follows:

**Dataset and image preprocessing:**

The Brain Tumor Segmentation (BraTS) challenge 2018 provided a dataset that contains 285 brain MRI 3D images of various modalities including native T1-weighted (T1) image, post-contrast T1-weighted (T1ce) image, T2-weighted (T2) image, T2-weighted Fluid-Attenuated Inversion Recovery (FLAIR) image, as well as expert-manually segmented labeling of 3 different types of abnormal regions of ET (GD-enhancing tumor), ED (peritumoral edema), and NCR/NET (the necrotic and non-enhancing tumor core). In the BrasTS 2018 challenge, we adopted individual T1ce and T2-weighted FLAIR images as primary input modalities in our experiments. Before constructing the final segmentation model for the competition, the intensity of the brain images was normalized as 0–255 based on the minimum and maximum values of the whole dataset. Then, we divided these 285 cases into 200/28/57 cases for the training/validation/testing set, respectively. The original dimension of each 3D MRI image is 240 × 240 pixels in transverse plane and 155 slices in height. After surveying the dataset, we cropped image dimension of the 3D images to 192 × 160 in transverse plane and 128 slices in height to discard the outer region which only contains non-brain information. The pixel value of segmented labeling image as ground-truth was 1, 2, 4, 0 for ET, ED, NCR/NET, and otherwise regions, respectively.

**Model Architecture:**

We designed two DL-based models based on 2D U-net architecture using Tensorflow 2.0 framework. The first model is called “Segmentor,” which used individual T1ce image and T2-weighted FLAIR image as input modalities and outputs the corresponding segmentation image of three different types of abnormal regions. The detailed model architecture of Segmentor was illustrated in the Supplementary Figure 1 which plotted with Tensorflow utilities for reproducibility. The concatenated T1ce image and T2-weighted FLAIR image passes through 43 neural network layers. All the Conv2D layers use 3 × 3 kernel and Rectified Linear Unit (ReLU) activation function, except for the last Conv2D layer, which uses Sigmoid activation function for the segmented output. The Batch Normalization layer, MaxPooling2D layer, and Dropout layer with dropout rate 50% are also implemented in our architecture.


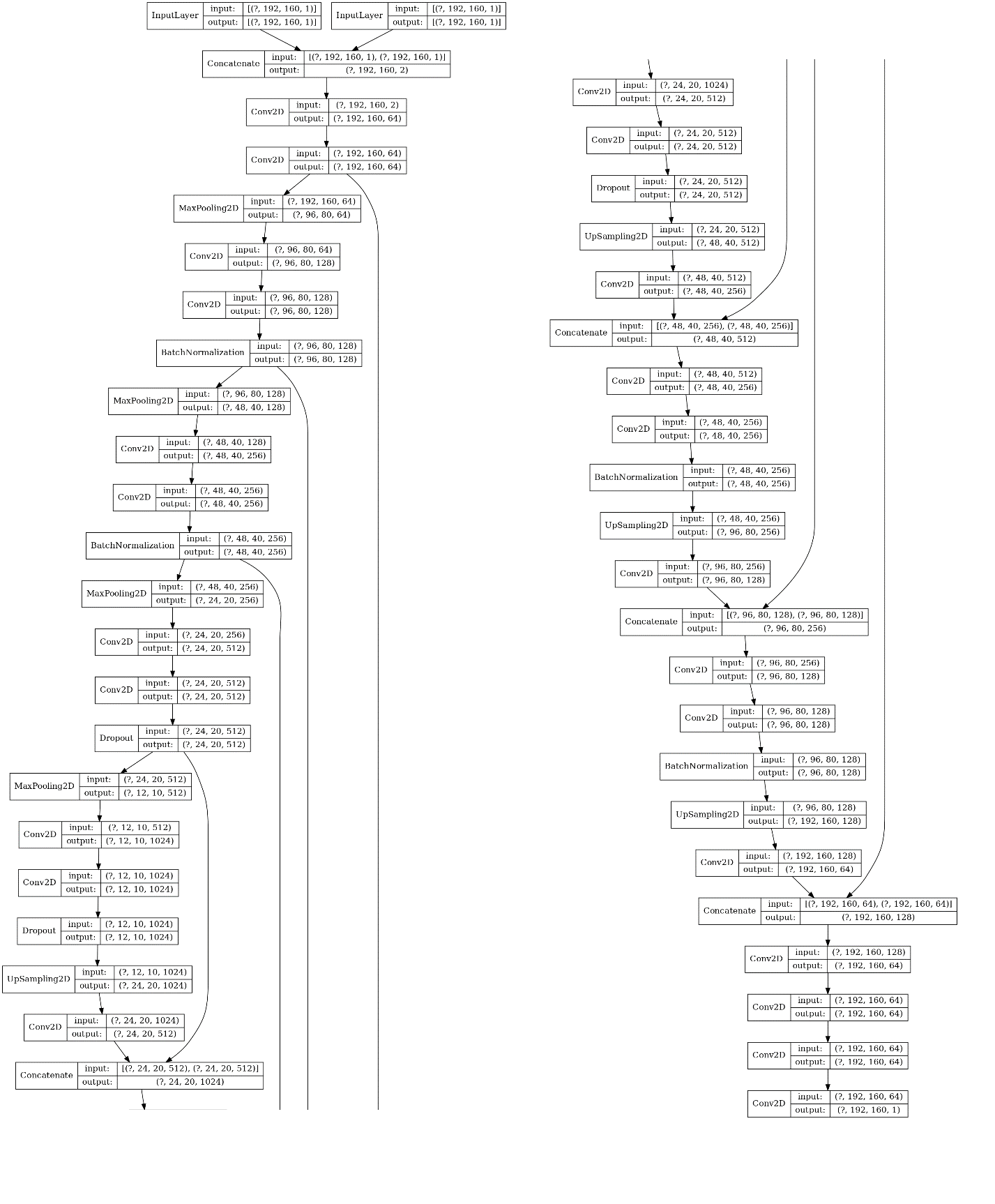


Supplementary Figure 1. Model Architecture of Segmentor

The second model is called “Enhancer,” which inputs only individual T1ce image and outputs an assisted T2-weighted FLAIR image which then feeds into the T2-weighted FLAIR image channel of the Segmentor (Supplementary Figure 2). The most important concept is that the “goal” of the Enhancer is not to generate an image similar to a traditional T2-weighted FLAIR image, but to generate an image which can be useful in achieving better segmented results for tumor localization. We fulfill this concept by adopting dice loss function for training both Segmentor and Enhancer. Because the Enhancer-generated image is fed into the T2-weighted FLAIR image channel of the Segmentor, we named the generated image “assisted T2-weighted FLAIR image.” For the model training process, we first trained the Segmentor with a batch of 32 image slices and then fixed the weights. Next, we trained the overall architecture and combined Enhancer and the Segmentor with the same batch. We repeated the previous two steps for 300 epochs. In the end, we combined every 2D image slice back to 3D volume to evaluate the segmentation performance. The optimizer for training the Segmentor and Enhancer is Adam with a learning rate of 0.001.


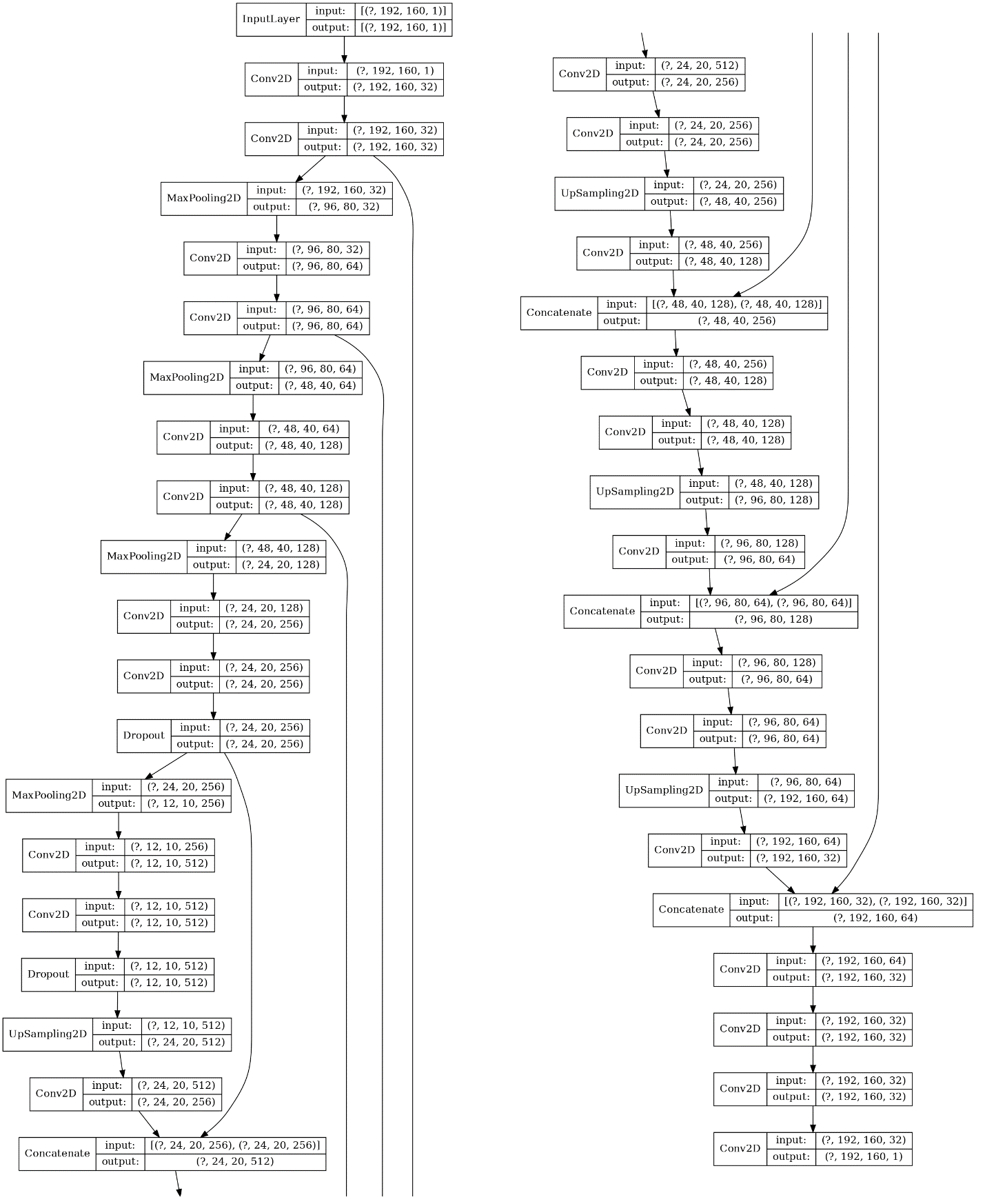


Supplementary Figure 2. Model Architecture of Enhancer
